# Mid-term outcomes of biventricular obstruction and left ventricular outflow tract obstruction after surgery correction in child and adolescent patients with hypertrophic cardiomyopathy

**DOI:** 10.1371/journal.pone.0192218

**Published:** 2018-02-06

**Authors:** Shanshan Zhai, Haitao Xu, Chaomei Fan, Yinjian Yang, Fei Hang, Xiying Guo, Hongyue Wang, Fujian Duan, Jun Yan

**Affiliations:** 1 Key Laboratory of Clinical Trial Research in Cardiovascular Drugs, Ministry of Health, Fuwai Hospital, National Center for Cardiovascular Diseases, Chinese Academy of Medical Sciences and Peking Union Medical College, Beijing, China; 2 Department of Pediatric Cardiovascular Surgery, Fuwai Hospital, National Center for Cardiovascular Diseases, Chinese Academy of Medical Sciences and Peking Union Medical College, Beijing, China; 3 Departments of Pathology, Fuwai Hospital, National Center for Cardiovascular Diseases, Chinese Academy of Medical Sciences and Peking Union Medical College, Beijing, China; 4 Department of Echocardiography, Fuwai Hospital, National Center for Cardiovascular Diseases, Chinese Academy of Medical Sciences and Peking Union Medical College, Beijing, China; Temple University, UNITED STATES

## Abstract

**Background:**

Data on the outcomes of hypertrophic cardiomyopathy (HCM) with biventricular obstruction are limited.

**Objective:**

Our aim is to compare mid-term outcomes of biventricular outflow tract obstruction (BVOTO) HCM, left ventricular outflow tract obstruction (LVOTO) HCM and nonobstructive hypertrophic cardiomyopathy (NO-HCM) in children and adolescents who were treated with standard medication or surgical resection.

**Methods:**

This retrospective study identified 21 BVOTO patients and recruited 27 LVOTO and 24 NO-HCM patients younger than 18 years presenting at our institution. The primary endpoint was all-cause death, and secondary endpoints were cardiovascular events.

**Results:**

More BVOTO patients (61.9%) than LVOTO (19.2%) and NO-HCM patients (25%) exhibited New York Heart Association (NYHA) III/IV status (p < 0.01). Fourteen BVOTO and 16 LVOTO patients obtained a significant reduction of outflow tract pressure gradients after surgery (vs. preoperative baseline, p < 0.001). One of the 14 BVOTO patients died, whereas no deaths occurred among LVOTO patients. Three of 14 BVOTO surgery patients had complete heart block (CHB) and 4 had new right bundle branch block (RBBB), while no CHB or RBBB occurred in the LVOTO surgery patients. The BVOTO patients had a longer duration of aortic cross-clamping and postoperative hospital days than the LVOTO patients (p < 0.05). During a median 42-month follow-up, no deaths occurred among the remaining patients. The primary and secondary endpoint-free survival rates of the BVOTO group were comparable to those of the LVOTO and NO-HCM groups.

**Conclusions:**

In children and adolescents, BVOTO patients were associated with more severe symptoms than LVOTO and NO-HCM patients; however, good mid-term outcomes similar to those of the LVOTO and NO-HCM groups can be achieved with the application of contemporary cardiovascular treatment strategies. Notably, BVOTO surgery was associated with an increased risk of CHB and RBBB compared to LVOTO surgery.

## Introduction

Hypertrophic cardiomyopathy (HCM) is a common inherited heart disease characterized by marked heterogeneity in phenotypic and genetic expression, clinical presentation, and natural history [1]. Originally, two phenotypes were considered: hypertrophic obstructive cardiomyopathy (HOCM) and non-obstructive hypertrophic cardiomyopathy (NO-HCM) [2]. Specifically, patients with HOCM have worse overall, HCM-related, and sudden cardiac death (SCD)-related survival than patients with NO-HCM[3], and left ventricular outflow tract obstruction (LVOTO) becomes an independent risk factor for SCD [4, 5].

Obstruction can occur in the left ventricular outflow tract (LVOT), mid cavity, apex, or right ventricular outflow tract (RVOT) due to apposition of the opposing walls [6]. However, because the right ventricle (RV) is ignored in cardiac examinations, there are limited data regarding the incidence, natural history, and treatments of HCM patients with the relatively rare RVOT obstruction (RVOTO). RVOTO is often accompanied by LVOTO, which causes biventricular outflow tract obstruction (BVOTO) [7]. Although both LVOTO and RVOTO are related to severe symptoms [8–12], there is no direct evidence that BVOTO patients have a less favourable prognosis than LVOTO patients.

BVOTO is relatively common in children and young adults and rare among elderly patients with HCM[13]. To prevent the bias of young age, which is identified as a risk factor for adverse events of HCM, patients with LVOTO and NO-HCM were screened and selected based on their similarity in age with BVOTO patients. The aim of this study was to compare mid-term outcomes of BVOTO and LVOTO in children and adolescents.

## Methods

### Study design and population

This retrospective study was approved by the research ethics boards of Fuwai Hospital and was conducted on May 1, 2016. From January 1994 to January 2016, 4945 patients were diagnosed with HCM at Fuwai Hospital, National Centre for Cardiovascular Diseases, Chinese Academy of Medical Sciences, and Peking Union Medical College. In children and adolescents, 21 BVOTO patients, 27 LVOTO patients, and 24 NO-HCM patients (control group) were matched based on gender and age within 5 years. In an adult, HCM is defined by a wall thickness ≥15 mm in one or more LV myocardial segments—as measured by any imaging technique (echocardiography, cardiac magnetic resonance imaging (CMR) or computed tomography (CT)).As in adults, the diagnosis of HCM in children requires an LV wall thickness more than two standard deviations greater than the predicted mean (z-score .2, where a z-score is defined as the number of standard deviations from the population mean) [5]. The LVOT and RVOT pressure gradients were measured using continuous-wave Doppler echocardiography, at rest and after provocative maneuvers. Patients were considered to have biventricular obstruction if the LVOT pressure gradient was ≥30 mm Hg and RVOT pressure gradient was ≥25 mm Hg at the same time[14, 15]. Patients with rheumatic valvular heart disease, double-chambered RV, pulmonary stenosis, or pulmonary arterial hypertension were excluded. Because this was not a clinical trial, parents or guardians of patients provided verbal informed consent for the use of their clinical information in this study. Their names and personal information were kept confidential. The data for each patient were not recorded in the HCM database unless the corresponding parents or guardians of patient agreed to participate in the study. This consent procedure was approved by ethics committee of Fuwai Hospital.

### Endpoints

The primary clinical endpoint was all-cause death. The secondary endpoints were cardiovascular-related events, including atrial fibrillation (AF), syncope, non-sustained ventricular tachycardia (NSVT; <30 seconds consecutive ventricular), progressive heart failure (HF) with an increase in New York Heart Association (NYHA) functional class ≥1, and embolic stroke [16].

### Data collection and follow-up

Demographic information, clinical information, electrocardiogram (ECG), Holter, echocardiography [left atrial dimension (LAD), left ventricular end-diastolic dimension (LVEDd), left ventricular ejection fraction (LVEF), systolic anterior motion (SAM), left ventricular outflow tract (LVOT) pressure gradient, right ventricular outflow tract (RVOT) pressure gradient, septal wall thickness (SWT), left ventricular mass index (LVMI), mitral regurgitation (MR), late gadolinium enhancement (LGE) of cardiac magnetic resonance (CMR) imaging data, histopathologic findings, and the operative notes were obtained at the initial visit. All patients were evaluated for the following characteristics: NYHA functional class, N-terminal pro-brain natriuretic peptide (NT-pro-BNP) level, and SCD risk factors (NSVT, maximal left ventricle (LV) wall thickness ≥30 mm, family history of SCD, unexplained syncope [1]). Follow-up started at the time of intervention and was assessed by subsequent clinic visits to the outpatient departments and telephone interviews with the patients and their relatives. In the medically treated cohort, follow-up started at the first outpatient clinic contact [17]. If no endpoints occurred during follow-up, the final censoring date was set at May 1, 2016.

### Statistical analysis

Normally distributed continuous data are expressed as the means ± SD and were compared using Student’s t tests, one-way analysis of variance, and post hoc comparisons when appropriate. Non-normally distributed data are summarized as medians (interquartile range [IQR]) and were compared by Kruskal-Wallis t-tests. Categorical variables are expressed as percentages and were compared by Chi-square tests, Fisher tests and Yates tests when appropriate. Kaplan-Meier survival curves were constructed to graphically represent survival in each group. Differences in survival were compared using the log-rank test. SPSS 24.0 Statistical Software (SPSS Inc., Chicago, IL) and Prism GraphPad 6.0 (GraphPad Software Inc., La Jolla, CA) were used for calculations and illustrations. All tests were two-sided, and p-values <0.05 were considered statistically significant.

## Results

### Baseline characteristics

Table 1 lists the baseline characteristics of all patients. Of the 72 patients (mean age 7.08 ± 5.85 y, 67.6% male) included in this study, the percentage of males and mean age were similar among the BVOTO, LVOTO, and NO-HCM groups (57.1%, 70.4% and 70.8%; p = 0.546; 5.5 ± 5.3 y, 6.4 ± 5.7 y, and 9.1 ± 5.9 y; respectively; p = 0.09). BVOTO patients manifested more serious symptoms, such as exertional dyspnoea, chest pain, syncope, palpitation and growth retardation than LVOTO and NO-HCM patients (81%, 48.1%, and 41.7%, p = 0.019) and had a higher prevalence of NYHA III/IV (61.9%, 19.2%, and 25%; p = 0.004) with worse diastolic function (E/A ratio > 2, 19%, 0, and 0; p = 0.006) and higher degree of NT-proBNP (4678 ± 1658, 2180 ± 1214, and 1238 ± 846; p < 0.001).

**Table 1:** Baseline characteristics.

Variables	BVOTO(n = 21)	LVOTO(n = 27)	NO-HCM(n = 24)
**Age, y**	5.5 ± 5.3	6.4 ± 5.7	9.1 ± 5.9
**Male, n (%)**	12 (57.1%)	19 (70.4%)	17 (70.8%)
**Symptoms, n (%)**	17 (81%)	13 (48.1%)	10 (41.7%)
**Dyspnoea, n (%)**	9 (42.9%)*	7 (25.9%)	3 (12.5%)
**Chest pain, n (%)**	2 (9.5%)	1 (3.7%)	4 (16.7%)
**Syncope, n (%)**	3 (14.3%)	3 (11.1%)	5 (20.82%)
**Palpitation, n (%)**	2 (9.5%)	1 (3.7%)	1 (4.2%)
**Growth retardation, n (%)**	2 (9.5%)	1 (3.7%)	0
**NYHA class III/IV, n (%)**	13 (61.9%)*#	5 (19.2%)	6 (25%)
**Murmur, n(%)**	7 (33.3%)	16 (59.3%) ‡	3 (12.5%)
**SBP, mmHg**	102.4±15.1	105.4±16.4	107.2±10.7
**DBP, mmHg**	60.4±7.4	58.2±10.2	62.6±8.1
**HR**	94.4±25.9*	95.9±21.7‡	78.6±16.9
**BSA, kg/m**^**2**^	1.05±0.56	1.11±0.59	1.27±0.53
**Concomitant disease, n (%)**	5 (23.8%)	2 (7.4%)	7 (29.2%)
**Atrial fibrillation, n(%)**	1 (4.8%)	0	2 (8.3%)
**Myocardial Bridge, n(%)**	1 (4.8%)	1 (3.7%)	1 (4.2%)
**ASD, n(%)**	3 (14.3%)	2 (7.4%)	2 (8.3%)
**PDA, n(%)**	1 (4.8%)	0	2 (8.3%)
**Echocardiography**			
**LVOT gradient (mmHg)**	70 (IQR, 50–103)§	77 (IQR,52–102)§	7 (IQR,4–8)
**RVOT gradient (mmHg)**	38 (IQR,32–71)§†	5 (IQR,4–7)	5 (IQR,4–6)
**Maximal SWT (mm)**	23.4 ± 8.6*	21.1 ± 9.0	18.5±6.1
**PWT (mm)**	10.9 ± 5.9	10.2 ± 4.2	10.2 ± 4.6
**LVMI (g/m**^**2**^**)**	207±137	170±80	165±92
**LAD, mm**	29±9.5	32±8.2	29±7.9
**LVEDd, mm**	31±7.9‡	32±8.4‡	40±11.5
**LVEF**	67.6±15.6	70.4±7.9*	63.8±10.1
**SAM positive, n (%)**	20 (90.5%)	24 (88.9%)	NA
**MR, n (%)**	14 (66.7%)§	18 (66.7%) §	2 (8.3%)
**E/A ratio < 1, n (%)**	9 (42.9%)	9 (33.3%)	8 (33.3%)
**E/A ratio > 2, n (%)**	4 (19%)*‖	0	0
**LGE** ^**a**^**, n(%)**	9 (75%)	7 (87.5%)	11 (84.6%)
**NT-proBNP**	4678±1658§†	2180±1214	1238±846
**Family history of HCM, n (%)**	2 (9.5%)	3 (11.1%)	4 (16.7%)
**Family history of SCD, n (%)**	2 (9.5%)	2 (7.4%)	3 (12.5%)
**SCD assessments**			
**≥1 risk factors, n(%)**	9 (42.9%)	8 (29.6%)	8 (33.3%)
**≥2 risk factors, n(%)**	3 (14.3%)	2 (7.4%)	1 (4.2%)
**Baseline Medications, n (%)**			
**Beta-blocker**	12 (57.1%)	15 (55.6%)	14 (58.3%)
**Calcium channel blocker**	2 (9.5%)	3 (11.1%)	4 (16.7%)
**Diuretic drugs**	10 (47.6%)	15 (55.6%)	7 (29.2%)

NYHA = New York Heart Associational; LVOT = left ventricular outflow tract; RVOT = right ventricular outflow tract; SWT = septal wall thickness; PWT = posterior wall thickness; LVMI = left ventricular mass index; LAD = left atrial dimension; LVEDd = left ventricular end-diastolic dimension; LVEF = left ventricular ejection fraction; SAM = systolic anterior motion; MR = mitral regurgitation; SCD = sudden cardiac death.

^a^ CMR was performed in 12 BVOTO, 8 LVOTO, and 13 NOHCM patients

§, p < 0.001

‡, p < 0.01

*, p < 0.05 compared with NO-CM

†, p < 0.001

#, p < 0.01

‖, p < 0.05 compared with LVOTO

BVOTO, LVOTO, and NO-HCM child and adolescent patients had similar incidences of concomitant diseases, such as AF, myocardial bridge (MB), atrial septal defect (ASD), and patent ductus arteriosus (PDA). Patients with BVOTO and LVOTO had increased incidences of MR (66.7%, 66.7%, and 8.3%; respectively; p < 0.001) and decreased LVEDd (31 ± 7.9, 32 ± 8.4, and 40 ± 11.5; p = 0.004) compared to NO-HCM patients. BVOTO patients had a thicker maximal SWT than NO-HCM patients (23.4 ± 8.6 vs. 18.5 ± 6.1; p = 0.04). No significant differences in the LA, LVEDd, LVEF, maximal septal wall thickness (SWT), LVMI, LVOT pressure gradient or prevalence of LGE were noted between the BVOTO and LVOTO patients, with the exception that BVOTO patients had a higher RVOT gradient and worse diastolic function than LVOTO patients.

The distribution of established risk factors for SCD among the 3 groups is presented in Table 1. Complete risk stratification was not available for all patients due to the lack of an abnormal blood pressure response. No significant differences in ≥1 or ≥2 established risk factors for SCD were noted between the BVOTO, LVOTO, and NO-HCM patients. Nearly half of the patients in the 3 groups received beta-blocker, calcium channel blocker and diuretic drugs.

### Operation data and postoperative results

Table 2 summarizes anatomical features, surgical procedures and postoperative early results.

**Table 2:** Operation data and postoperative results.

Variables	BVOTO (n = 14)	LVOTO(n = 16)
**LV pattern of obstruction**		
**Sub-aortic alone, n (%)**	10 (71.4%)	10 (62.5%)
**With MidV, n (%)**	1 (7.1%)	2 (12.5%)
**With AMB, n (%)**	3 (21.4%)	4 (25%)
**RV pattern of obstruction**		
**Septal alone, n (%)**	3 (21.4%)	NA
**With Infundibular, n (%)**	5 (35.7%)	NA
**With AMB, n (%)**	6 (42.8%)	NA
**With Free Wall, n (%)**	3 (21.4%)	NA
**Procedural details**		
**LV myectomy**	14	16
**TAortic alone, n (%)**	11 (78.5%)	11 (68.7%)
**With AMB resection, n (%)**	3 (21.4%)	5 (31.3%)
**RV surgery**	11	NA
**Septal resection alone**	3 (27.3%)	NA
**With infundibular resection, n (%)**	3 (27.3%)	NA
**With AMB resection, n (%)**	5 (45.4%)	NA
**With free Wall resection, n (%)**	5 (45.4%)	NA
**With RVOT patch, n (%)**	2 (18.1%)	NA
**Concomitant procedures, n (%)**	6 (42.9%)	3 (18.8%)
**CABG, n (%)**	1(7.1%)	0
**Mitral valvuloplasty, n (%)**	2 (14.2%)	1 (6.3%)
**Atrial septal defect repair, n (%)**	2 (14.2%)	3 (18.8%)
**PDA repair, n (%)**	1 (7.1%)	0
**Preoperative LVOT gradient (mmHg)**	69 (IQR, 51–99)	83 (IQR, 64–105)
**Preoperative RVOT gradient (mmHg)**	38 (IQR, 31–87)	5 (IQR, 4–8)
**Last residual LVOT gradient (mmHg)**	9 (IQR, 4–21) ^	15 (IQR, 5–20) ^
**Last residual RVOT gradient (mmHg)**	12 (IQR, 6–25) #^	4 (IQR, 4–6)
**SAM positive, n (%)**	0 ^&^	0 ^&^
**MR positive, n (%)**	0^&^	3 (18.8%)^&^
**Periprocedural death**	1	0
**Periprocedural complications**		
**Complete heart block, n (%)**	3 (21.4%)	0
**New RBBB, n (%)**	4 (28.6%) ‖	0
**New LBBB, n (%)**	2 (14.3%)	3 (18.8%)
**Duration of cardiopulmonary bypass (min)**	119 (IQR, 102–157)	92 (IQR, 71–127)
**Duration of aortic cross clamping (min)**	77 (IQR, 54–92) ‖	57 (IQR, 39–67)
**Duration of postoperative ventilation (h)**	14.5 (IQR, 12.0–20)	15.6 (IQR, 12.1–19.2)
**Postoperative hospital stay (d)**	13 (IQR, 7–18) ‖	7 (IQR, 7–9)
**Latest echocardiography**		
**Maximal SWT (mm)**	15.5±7.2^	15.4±6.4^
**PWT (mm)**	11.8±6.2	10.8±3.8
**LAD, mm**	26.2±7.5^&^	27.2±5.3^
**LVEDd, mm**	31.5±8.0	36.5±9.3

MidV: midventricular; AMB: Anomalous muscular bundles; TAortic: trans-aortic; CABG, Coronary artery bypass grafting; PDA: patent ductus arteriosus RBBB, right bundle branch block; LBBB, left bundle branch block; VSD, ventricular septal defect.

#, p < 0.01

‖, p < 0.05 compared with LVOTO

^, p < 0.01

^&^, p < 0.05 compared with preoperative measurements

#### Anatomic features

The mechanisms of obstruction were reviewed using operative notes, echocardiography and CMR. The presence of septal hypertrophy involving the subaortic region alone was the most common factor contributing to obstruction in the left side in both the BVOTO and LVOTO groups (71.4% and 62.5%), followed by the presence of abnormal papillary muscles (AMB) (21.4% and 25%) and extensive septal hypertrophy extending to the midventricular level (7.1% and 12.5%). In the BVOTO group, 3 patients had right-sided septal hypertrophy bulging into the RVOT as an isolated cause of obstruction in the right side, while septal muscle bundles in the right ventricle were noted in 6 patients, septal hypertrophy extending to the infundibular level occurred in 5 patients and 3 patients had right ventricular free wall hypertrophy.

#### Surgical procedures

Based on the anatomic features, biventricular myectomy was performed in 11 of the 14 BVOTO patients while 3 patients with projection of hypertrophied RV septum into the RV cavity alone and 16 LVOTO patients had left-sided ventricular resection. In the 11 patients who underwent biventricular resection, all of them had left ventricular resection, regard to the right ventricular surgical procedures, 3 of them had right ventricular septal resection alone, and 8 patients had additional operations with septal resection, including infundibular resection (n = 3), AMB resection (n = 5), free wall resection (n = 5), and RVOT patch (n = 2). Of the 11 patients, 4 had 2 additional procedures and 1 had 3 additional procedures. In addition to trans-aortic left ventricular septal resection in both the BVOTO and LVOTO groups, AMB resection was performed in some of the patients (21.4% in BVOTO and 31.3% in LVOTO). The durations of aortic cross-clamping and postoperative hospital stay were longer in the BVOTO group than the LVOTO group. However, the duration of cardiopulmonary bypass and postoperative ventilation was comparable between the two groups. After thorough preoperative assessment and careful intraoperative investigation, 42.9% of the BVOTO patients and 18.8% of the LVOTO patients underwent concomitant procedures (p = 0.236).

#### Postoperative early results

In the BVOTO group, a 1-year-old boy died of pneumonia on the 22nd day after the procedure despite the administration of extracorporeal membrane oxygenation (ECMO) therapy. No periprocedural deaths occurred in the LVOTO group. In the first 30 post-procedure days, new right bundle branch block (RBBB) occurred more frequently in the BVOTO group than the LVOTO group (n = 4 [28.6%] vs. n = 0 [0%], p = 0.036), and BVOTO surgery tended to increase the prevalence of new complete heart block (CHB) more than LVOTO surgery but without statistical significance (n = 3 [21.4%] vs. n = 0 [0%], p = 0.098); the incidences of new left bundle branch block (LBBB) were similar between the BVOTO and LVOTO groups (n = 2 [14.3%] vs. n = 3 [18.8%], p = 1). No iatrogenic ventricular septal defects or moderate-to-severe iatrogenic aortic regurgitation occurred after the operation.

The LVOT gradient was reduced from a median of 69 mmHg (IQR: 51 to 99 mmHg) to 9 mmHg (IQR: 4 to 21 mmHg) in the BVOTO group and from a median of 83 mmHg (IQR: 64 to 105 mmHg) to 15 mmHg (IQR: 5 to 20 mmHg) in the LVOTO group. The RVOT gradient was reduced from a median of 42 mmHg (IQR: 33 to 90 mmHg) to 10 mmHg (IQR: 4 to 16 mm Hg) after biventricular resection and from a median of 32 mmHg (IQR: 25 to 42 mmHg) to 9 mmHg (IQR: 7.8 to 16 mm Hg) after left-sided ventricular resection alone in the BVOTO group. Systolic anterior motion (SAM) was eliminated in both BVOTO groups, and MR was alleviated in BVOTO group (85.7% vs 0%, p < 0.001) and LVOTO group (87.5% vs 18.8%, p < 0.001) after surgery.

**Fig 1:**
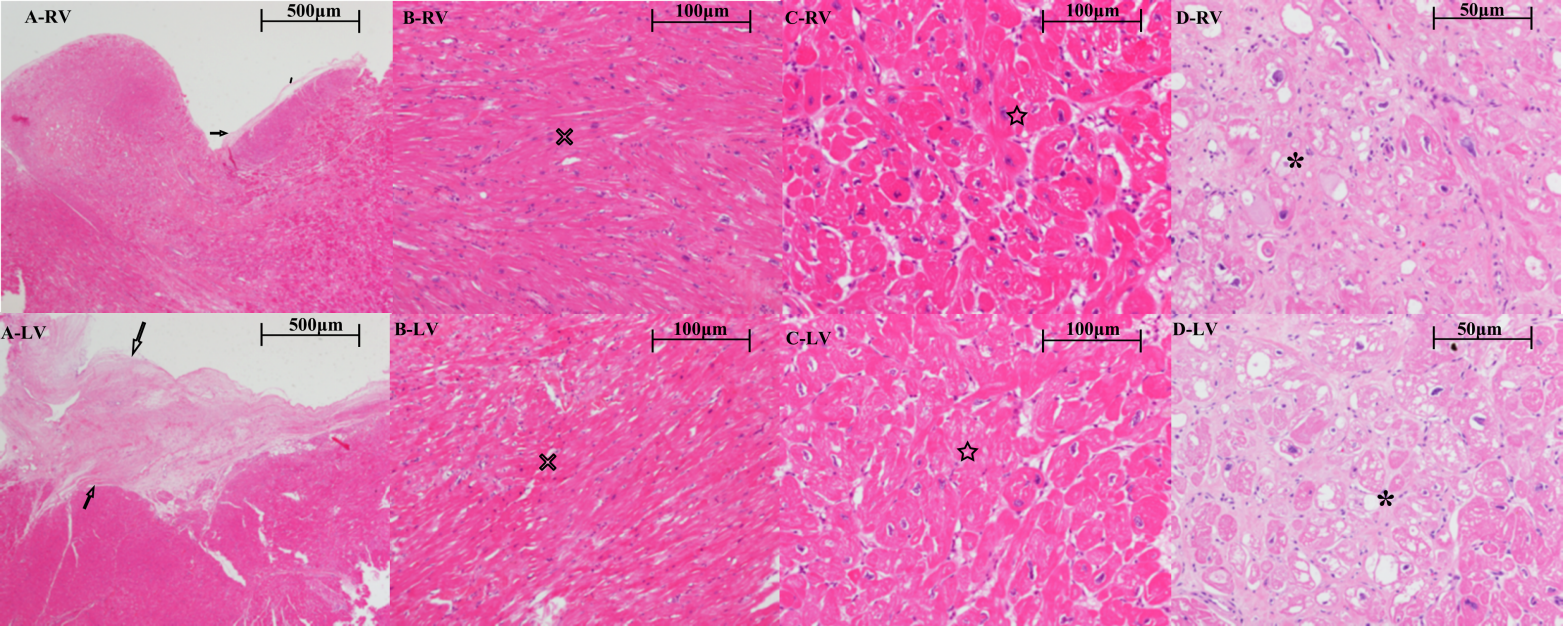
Microscopic findings of BVOTO. The (A-RV) and (A-LV) images reveal a higher degree of endocardium incrassation in the left ventricle (LV) than the right ventricle (RV) (black arrows). The (B-RV) and (B-LV) images demonstrate more serious cardiomyocyte disarray in the RV than the LV. No significant differences in cardiomyocyte hypertrophy (C-RV vs. C-LV) or vacuolization (D-RV vs. D-LV) were noted between the RV and LV.

A significantly higher degree of endocardium incrassation was observed in the LV than in the RV, and a higher degree of cardiomyocyte disarray was observed in the RV than the LV in the BVOTO group. No significant differences in cardiomyocyte hypertrophy or vacuolization were noted between the RV and LV (Fig 1).

### Follow-up

**Fig 2:**
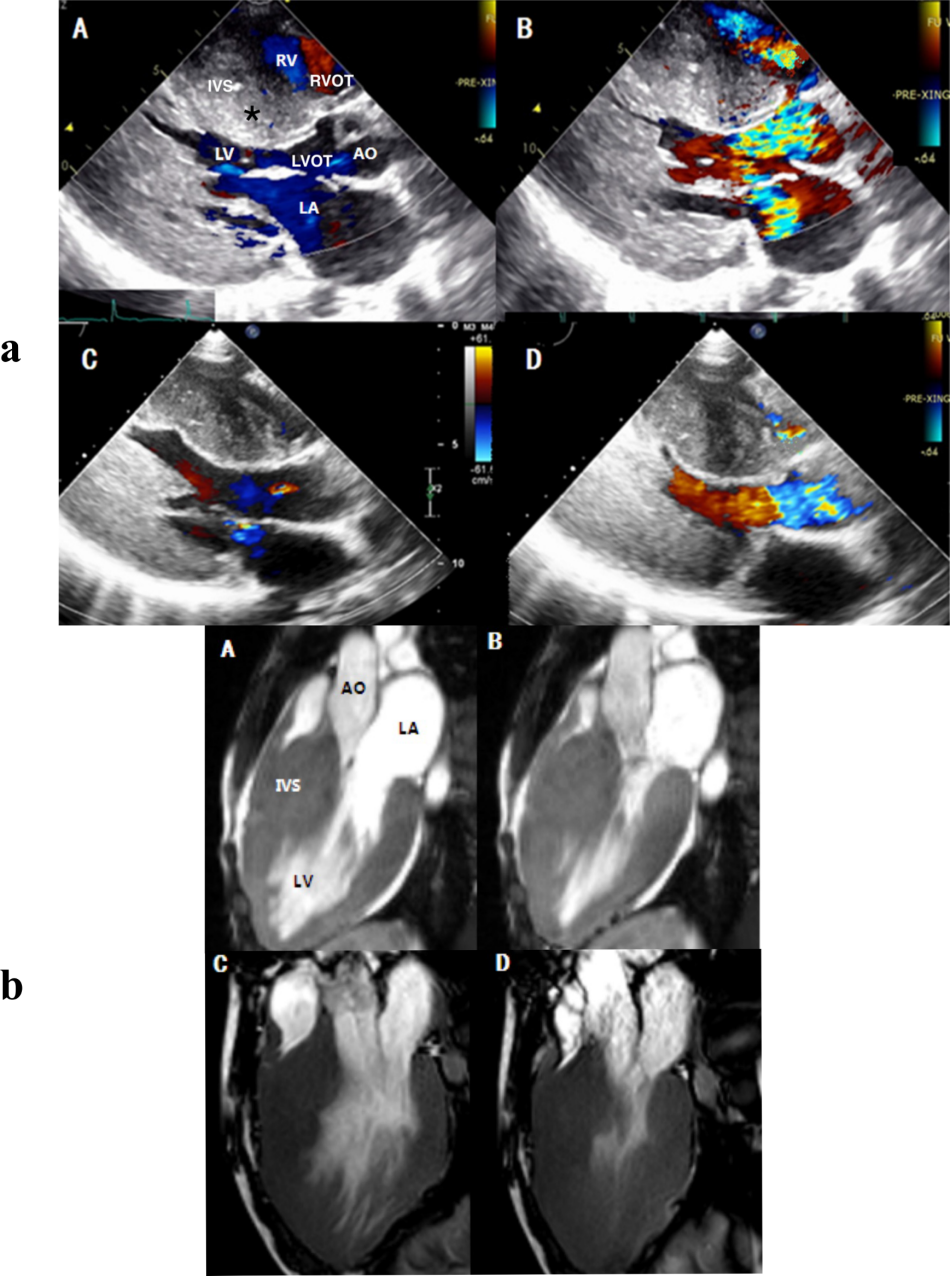
Fig 2a. Preoperative two-dimensional transthoracic echocardiography (tte) parasternal long axis (PLAX) views in a 16-year-old hypertrophic cardiomyopathy patient with BVOTO. (A) PLAX view demonstrating the massive septal hypertrophy and the thickening of the ventricular septum bulging into the LVOT and RVOT resulting in biventricular obstructions (the colour flows). (B) Colour Doppler flow imaging of PLAX view during systole showing high velocity jet flow simultaneously in both LVOT and RVOT. Postoperative PLAX views showing a substantial decrease in the ventricular septum thickness and an increase in the RV and LV cavity sizes during diastole (C) and the LV and RV colour flows showing laminar without evidence of significant residual obstructions during systole (D).RV: right ventricle; RVOT: right ventricular outflow tract; IVS: interventricular septum; LV: left ventricle; LA: left atrium; LVOT: left ventricular outflow tract.AO: aorta. Fig 2b. Preoperational cardiovascular magnetic resonance (CMR) image 3-chamber views during diastole (A) and systole (B) showing remarkable myocardial hypertrophy at the base ventricular level with LVOT and RVOT obstruction. The postoperative CMR images (C, D) showing thinner IVS, wider LVOT and RVOT diameter and larger LV and RV cavity without the projection of septum into RVOT or LVOT after biventricular resection. LA: left atrial; LV: left ventricular.

During a median 42-month follow-up, no deaths occurred among the remaining patients(n = 71). At the latest follow-up, all patients in the BVOTO (n = 13) and LVOTO(n = 16) surgery groups exhibited significant improvements in NYHA functional class with a substantial reduction in maximal septal thickness, LA diameter, and residual LVOT and RVOT gradients (Fig 2) (Tables 2 and 3).

**Table 3:** Middle-Term results in the three groups.

Variables	BVOTO (n = 21)	LVOTO (n = 27)	NO-HCM(n = 24)
**Follow-up Months**	26.9±19.7*	40.6±36.3	55.9±25.3
**Death**	1	0	0
**Cardiovascular events (%)**	4 (19%)	1 (3.8%)	3 (12.5%)
**Atrial fibrillation, n (%)**	3 (14.3%)	1 (3.7%)	3 (12.5%)
**NSVT, n (%)**	1 (4.8%)	0	0
**Latest NYHAIII/IV, n (%)**	0	3 (11.1%)	1 (4.2%)

Abbreviations as in Table 1 & Table 2

*, p < 0.05 compared with NO-HCM

**Fig 3:**
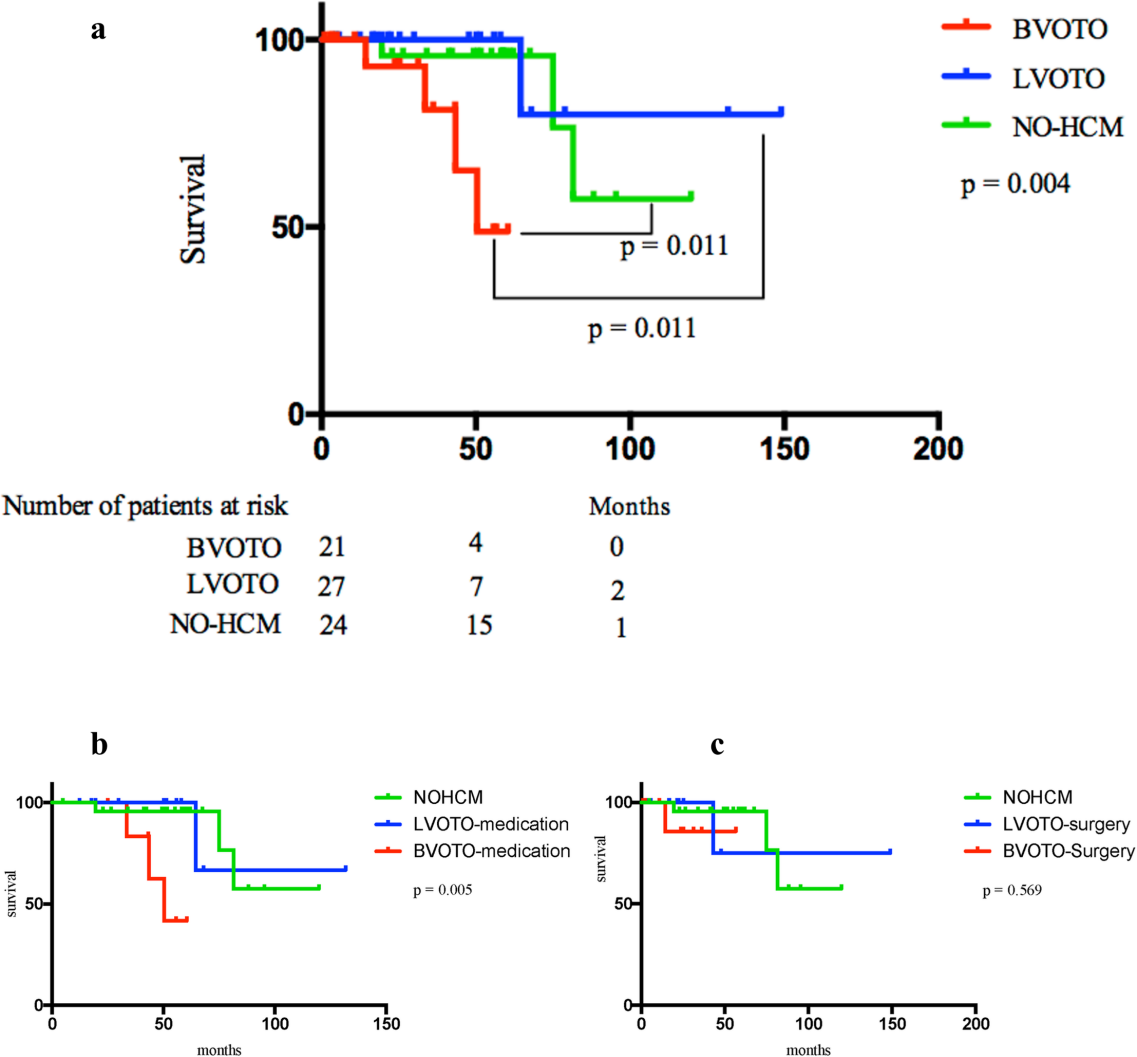
Fig 3a. Kaplan-Meier estimates of cardiovascular event-free survival among the entire BVOTO, LVOTO and NO-HCM groups; Fig 3b. Kaplan-Meier estimates of cardiovascular event-free survival among the BVOTO medication, LVOTO medication and NO-HCM groups; Fig 3c. Kaplan-Meier estimates of cardiovascular event-free survival in the BVOTO surgery, LVOTO surgery and NO-HCM groups.

The morbidity of cardiovascular events was similar among the BVOTO(n = 20), LVOT(n = 27), and NO-HCM(n = 24) groups (19%, 3.8%, and 12.5%; p = 0.236), and the leading causes of cardiovascular events were AF and NSVT. Kaplan-Meier estimates demonstrated that the secondary endpoint-free survival rates of all BVOTO patients were lower than those of the entire LVOTO and NO-HCM groups (log-rank p = 0.004, Fig 3). The BVOTO medication group was associated with less favourable outcomes than the LVOTO medication and NO-HCM groups (log-rank p = 0.005, Fig 3B). However, the secondary endpoint-free survival rates were similar among the BVOTO surgery, LVOTO surgery, and NO-HCM groups (log-rank p = 0.569, Fig 3C). For the total study population, the RVOT pressure gradient (HR, 1.042; 95% CI, 1.005–1.080, p = 0.028) and age (HR, 1.154; 95% CI, 1.022–1.303, p = 0.021) were independent predictors of the secondary endpoint. (Fig 3)

## Discussion

RVOTO remains a rare finding (15% of HCM patients [18]) despite the understanding of morphological abnormalities in RV wall thickness and mass in HCM patients [7]. BVOTO HCM is substantially rarer. The prevalence of BVOTO accounted for 0.42% of the entire HCM population, which is less than the data reported by Quintana, Johnson (19). However, mild-to-moderate right-sided obstruction is commonly overlooked, and the prevalence of right-sided pathology may be underestimated by routine examinations [20].

The anatomical aspects of RV involvement in HCM were initially described by Maron, Hauser (10). In contrast to the dynamic lesions occurring in LVOTO, including asymmetrical septal hypertrophy, SAM of the mitral valve, mitral apparatus-related anomalies, and apex-IVS muscle bundles [7], obstruction in the RV is due to static and fixed impediments to RV outflow, such as the projection of a hypertrophied RV infundibulum or septum into the RV cavity, free wall hypertrophy, abnormal muscular bundles (AMB), [12, 21]. The BVOTO patterns in our cohort were similar to those reported by Quintana, Johnson (19).

Previous studies demonstrate that LVOTO is a risk factor for premature death [22], and RVOTO is related to severe symptoms [8–12]. The combination of LVOTO and RVOTO can cause lethal haemodynamic changes in HCM patients [15]. Several studies have reported BVOTO cases. Most of these cases occurred in children or infants, and the mortality rate was relatively high [12, 13, 23, 24]. In our cohort, patients with BVOTO were 5.5 ± 5.3 y of age, which is substantially younger than HCM patients overall; these patients also presented more advanced NYHA functional class (III/IV) requiring surgical intervention than the only LVOTO patients. This indicates that BVOTO is a serious and advanced hypertrophic process involving both ventricles with a higher degree of cardiomyocyte disarray and higher level of NT-proBNP. Current thinking suggests that in contrast to patients with LVOTO in whom SCD was more common than death due to progressive heart failure, in patients with BVOTO, SCD was less common than death due to progressive congestive heart failure.[13]. Our findings were consistent with the previous studies. Moreover, BVOTO patients were associated with increased incidences of diastolic dysfunction. This finding implies that the LV diastolic dysfunction of BVOTO patients contributes to progressive congestive heart failure. In addition to traditional risk factors, RVOTO was an independent risk factor of cardiovascular-related morbidity in our study.

Given the low incidence and survival rate, treatments for BVOTO are not well established. In 1983, Maron and Tajik [13] reported that two of three BVOTO patients died shortly after the resection of right ventricular outflow tract muscle. In 1993, Maron and McIntosh [12] recorded three of the four BVOTO patients underwent biventricular resection; one patient died soon after the operation, and another patient died 7 days after the Morrow procedure alone. In 1996, McCully and Nishimura [23] documented the death of a 26-year-old woman with a severe BVOTO after biventricular septal resection. Theodoro and Danielson [24] described a 2-month-old girl with BVOTO HCM who did not improve after biventricular resection and underwent heart transplantation. The previous works indicated that biventricular resection was ineffective and associated with a high risk of death. However, biventricular resection was successfully performed in a 54-year-old woman (LVIPG = 150 mmHG, RVIPG = 130 mmHG) [25] and a 57-year-old man (LVIPG = 109 mmHg, RVIPG = 138 mmHg) [26] with lasting outcomes. Quintana, Johnson [19] recently indicated that relief of BVOTO could be achieved with low mortality and good long-term outcomes via biventricular resection in children and young adults. Our study confirmed that surgery could abolish BVOTO with a relatively lower risk of perioperative mortality than before and result in better mid-term outcomes than for BVOTO patients who took medication and with similar outcomes as the age-matched NO-HCM patients. This improvement is due to several factors, including increased understanding of the condition, the introduction of intraoperative echocardiography, and improved myocardial preservation techniques and postoperative care [27].

In the respect of operations, 11 BVOTO patients underwent biventricular resection while 3 BVOTO patients had left-sided ventricular resection alone. The surgical types depend on the mechanisms and pressure gradient of RVOT obstruction, and age of surgery. RVOTO in the biventricular resection group were more commonly due to infundibular hypertrophy, free wall hypertrophy and AMB, which required right ventricular resection [10]. However, in the left ventricular resection group, RVOTO was attributed to the projection of hypertrophied RV septum into the RV cavity alone, which could be resolved by left-sided ventricular resection alone [21, 28]. On the basis of different RVOTO mechanisms, RVOT pressure gradient tended to be a bit lower in BVOTOT patients who had left-sided ventricular resection than those who underwent biventricular resection, even though without significance. And RVOT obstruction may be alleviated with growth and ageing, which would result in an increase in the size of the RVOT [13], the increased age of the patients in the left ventricular resection group than biventricular resection group (10 ± 6 y vs 4 ± 3 y) may contribute to the identical effect of the two operations.

However, compared to the LVOTO group, BVOTO patients had increased incidences of CHB and RBBB and longer aortic cross-clamping time and postoperative hospital stay with more resection sites (increased proportion of right ventricular septal resection). Prior studies showed that patients with complete RBBB preoperatively may have an increased risk of CHB after myectomy [29]. In this retrospective study, none of the 3 post-CHB patients had a previous history of complete RBBB; however, with similar incidences of new LBBB between the LVOTO and BVOTO surgery group, biventricular resection harmed the extra right bundle branch, which resulted in a higher prevalence of CHB. Therefore, it is necessary to note that biventricular resection imparts more severe damage to the cardiac conduction system in BVOTO patients than the Morrow procedure in LVOTO patients.

Alternative treatments in addition to surgery for BVOTO HCM patients were described in some cases, including cibenzoline [30, 31] and dual chamber pacing (DDD) implantation [32].

## Study limitations

This study was retrospective and nonrandomized. It was limited by the short follow-up and could not demonstrate long-term improvements. In our study, myectomy in children and adolescents was performed by only one surgeon, Jun Yan, with a high level of expertise. Therefore, the outcome cannot be easily extrapolated to other centres.

## Conclusions

In children and adolescents, BVOTO patients were associated with more severe symptoms than those in LVOTO and NO-HCM patients; however, good mid-term outcomes similar to those of the LVOTO and NO-HCM groups can be achieved with the application of contemporary cardiovascular treatment strategies. Notably, BVOTO surgery was associated with an increased risk of CHB and RBBB compared to LVOTO surgery.

## Supporting information

S1 Dataset Mid-term outcomes of BVOTO, LVOTO and NOHCM in child and adolescent patients with hypertrophic cardiomyopathy. (XLSX)

## Abbreviations

BVOTO: biventricular outflow tract obstruction
CHB: complete heart block
HCM: hypertrophic cardiomyopathy
LVOTO: left ventricular outflow tract obstruction
NYHA: New York Heart Association
NO: HCM, non-obstructive hypertrophic cardiomyopathy
PG: pressure gradient
RVOTO: right ventricular outflow tract obstruction
SCD: sudden cardiac death
